# Golden-rod Killing Horses

**Published:** 1896-02

**Authors:** J. L. Scott

**Affiliations:** State Veterinarian, Wisconsin


### GOLDEN-ROD KILLING HORSES.

By J. L. SCOTT, V.S.,
State Veterinarian, Wisconsin.

An article under the above heading has appeared in several newspapers. If I may venture to trench upon your valuable space, perhaps the presentation of the facts of the matter may not be uninteresting to your readers.

During the past four years a large number of horses have died in the northern part of this State from the ravages of a disease which has baffled the skill of veterinarians, and I have been called upon to investigate the matter as to the cause and nature of the malady. At first it was thought to be anthrax, and samples of the blood and sections from the spleen and other internal organs were sent to the Bureau of Animal Industry and to Dr. Russell, of the State University, for bacteriological examination. Numerous bacteria were found, but the bacillus anthrax was not present. The horses affected were, in the majority of cases, heavy draft-horses from the lumber-camps. These animals were brought from the woods in the spring, usually in good condition, and turned out to pasture. Most of them were fed grain while on pasture.

On the farm of Mr. C. F. Reynolds, Hayward, Wis., over seventy horses have died during the past four years from this peculiar malady. The pasture contained about four hundred acres, three hundred acres of which had been broken and seeded to timothy. Adjoining this was one hundred acres of "slashings," or land from which the timber had been cut but which had never been broken. This was thickly covered with golden-rod. On one side of the farm is a lake with a clean gravel bottom and shore. The lake is fed by springs. There is no marsh or low land on the farm. Upon investigation, I became convinced that the cause of the trouble was to be found either in the food or water, and watched the horses closely for several days and saw them eating the golden-rod greedily, some of them, especially those affected, seeming to prefer the plant to anything else.

I also visited the farm of Peter Truax, near Eau Claire. There is no golden-rod to be found on this farm, and the disease has not made its appearance. During the past summer Mr. Truax placed ten horses in pasture near by where the plant was plentiful, and eight of them died during the summer and the remaining two are affected. When the healthy horses are taken from the pasture in the fall the disease disappears. None of the animals attacked by the malady have recovered, and medicinal treatment does not seem to produce any beneficial effect.

*Symptoms.* The animal appears dull, ears drooped, temperature elevated, ranging from 103° to 107° F., during the entire course of the disease. The visible mucous membranes are pallid. On the mucous membranes of the vulva small petechial spots are seen. Occasionally the legs swell and œdematous enlargements appear under the abdomen. The appetite remains fairly good during the entire course of the disease. Emaciation takes place rapidly as the disease advances. Loss of coördination, with staggering gait. Death takes place in from two weeks to two months from the onset.

*Post-mortem.* On cutting open the body the blood appears to be completely disintegrated, resembling ordinary blood-serum. Intestines bloodless, with numerous petechial spots on the mucous membrane. Spleen enlarged, weighing from six to ten pounds. No structural changes apparent to the naked eye. The lungs and kidneys apparently normal. The brain and spinal cord were not examined.

I am fully convinced that this disease is due to either some poisonous principle in the plant, or some parasitic fungus upon the surface of the same. It is now too late in the season for any investigation to be carried on in this direction this year, but I intend to have the matter thoroughly investigated next summer.

---

AT the January meeting of the New York County Society, two veterinarians made the statement that a physic followed by two-drachm doses of citrate of lithia cured 70 per cent. of their cases of azoturia; also, that they have never noticed azoturia on sunshiny days.

THE Veterinary Department of Harvard has, according to the Boston *Herald* of January 1st, decided to establish a free clinic in connection with the hospital of the Veterinary Department. A new building has been obtained on Northampton Street, and is now being fitted up for this purpose. This plan has been completed through the assurance of two thousand dollars per year, for three years, by a number of Boston's public-spirited citizens. The new building is 60 by 100 feet, and one story in height, and its light and ventilation are said to be all that can be desired. The free clinic established will only be extended to those who cannot afford to pay professional charges for skilled work. Dr. P. J. Cronan, Assistant Surgeon and Instructor, will be in special charge of this establishment. Dr. Cronan is a graduate of the school, and has also had a post-graduate course in foreign schools.

The Veterinary Department has in the three classes this year sixty students with a graded course of nine months each.

